# Two decades of endemic dengue in Bangladesh (2000–2022): trends, seasonality, and impact of temperature and rainfall patterns on transmission dynamics

**DOI:** 10.1093/jme/tjae001

**Published:** 2024-01-22

**Authors:** Mohammad Nayeem Hasan, Ibrahim Khalil, Muhammad Abdul Baker Chowdhury, Mahbubur Rahman, Md Asaduzzaman, Masum Billah, Laila Arjuman Banu, Mahbub-Ul Alam, Atik Ahsan, Tieble Traore, Md Jamal Uddin, Roberto Galizi, Ilaria Russo, Alimuddin Zumla, Najmul Haider

**Affiliations:** Department of Statistics, Shahjalal University of Science and Technology, Sylhet 3114, Bangladesh; Department of Livestock Services, Ministry of Fisheries and Livestock, Bangladesh, Dhaka, Bangladesh; Department of Neurosurgery, University of Florida College of Medicine, Gainesville, FL 32610, USA; The Royal Veterinary College, University of London, Hawkshead Lane, North Mymms, Hatfield, Hertfordshire, UK; Institute of Epidemiology, Disease Control and Research (IEDCR), Ministry of Health and Family Welfare, Mohakhali, Dhaka, Bangladesh; School of Digital, Technologies, and Arts, Staffordshire University, Stoke on Trent ST4 2DE, UK; School of Digital, Technologies, and Arts, Staffordshire University, Stoke on Trent ST4 2DE, UK; Department of Anatomy, Bangabandhu Sheik Mujib Medical University, Dhaka, Bangladesh; Environmental Intervention Unit, International Centre for Diarrhoeal Diseases Research, Bangladesh (ICDDR,B), Dhaka 1212, Bangladesh; Environmental Intervention Unit, International Centre for Diarrhoeal Diseases Research, Bangladesh (ICDDR,B), Dhaka 1212, Bangladesh; Emergency Preparedness and Response Programme, WHO Regional Office for Africa, Dakar Hub, Dakar, Senegal; Department of Statistics, Shahjalal University of Science and Technology, Sylhet 3114, Bangladesh; Department of General Educational and Development, Daffodil International University, Dhaka, Bangladesh; School of Life Sciences, Faculty of Natural Sciences, Keele University, Keele, Staffordshire ST5 5BG, UK; School of Medicine, Faculty of Medicine and Health Sciences, Keele University, Staffordshire ST5 5BG, UK; Division of Infection and Immunity, Centre for Clinical Microbiology, University College London and NIHR-BRC, University College London Hospitals, London, UK; School of Life Sciences, Faculty of Natural Sciences, Keele University, Keele, Staffordshire ST5 5BG, UK

**Keywords:** dengue, Bangladesh, climate change, temperature, rainfall

## Abstract

The objectives of this study were to compare dengue virus (DENV) cases, deaths, case-fatality ratio [CFR], and meteorological parameters between the first and the recent decades of this century (2000–2010 vs. 2011–2022) and to describe the trends, seasonality, and impact of change of temperature and rainfall patterns on transmission dynamics of dengue in Bangladesh. For the period 2000–2022, dengue cases and death data from Bangladesh’s Ministry of Health and Family Welfare’s website, and meteorological data from the Bangladesh Meteorological Department were analyzed. A Poisson regression model was performed to identify the impact of meteorological parameters on the monthly dengue cases. A forecast of dengue cases was performed using an autoregressive integrated moving average model. Over the past 23 yr, a total of 244,246 dengue cases were reported including 849 deaths (CFR = 0.35%). The mean annual number of dengue cases increased 8 times during the second decade, with 2,216 cases during 2000–2010 vs. 18,321 cases during 2011–2022. The mean annual number of deaths doubled (21 vs. 46), but the overall CFR has decreased by one-third (0.69% vs. 0.23%). Concurrently, the annual mean temperature increased by 0.49 °C, and rainfall decreased by 314 mm with altered precipitation seasonality. Monthly mean temperature (Incidence risk ratio [IRR]: 1.26), first-lagged rainfall (IRR: 1.08), and second-lagged rainfall (IRR: 1.17) were significantly associated with monthly dengue cases. The increased local temperature and changes in rainfall seasonality might have contributed to the increased dengue cases in Bangladesh.

## Introduction

Dengue fever is a mosquito-borne disease (MVD) caused by 4 distinct serotypes of the dengue virus (DENV) within the family *Flaviviridae* (Simmonds et al. 2017). DENV is transmitted to humans by bites of *Aedes aegypti* (L.) and *Aedes albopictus* (Skuse) (WHO 2009, CDC 2019). DENV is endemic in over 125 countries, and the number of cases globally reported to WHO continues to increase yearly (Bhatt et al. 2013, WHO 2023). Annually, an estimated 390 million dengue infections are estimated worldwide, including 96 million clinical cases making DENV one of the most important vector-borne diseases (VBDs) (Murray et al. 2013, Messina et al. 2019, WHO 2023). Most infections (>80%) are self-limiting with no or mild clinical manifestation resulting in lifelong immunity for that serotype (WHO-Bangladesh 2022). However, reinfection with different serotypes, known as secondary or tertiary dengue infection, may result in severe dengue with an increasing risk of fatal outcome (Teo et al. 2023).

Currently, South and Southeast Asia are “hotspots” of DENV infection, with more than 50% of cases recorded in these regions (WHO South-East Asia 2023). The first DENV outbreak in Bangladesh was reported in 2000, and since then, dengue has become endemic in the country posing a significant health challenge (Sharmin et al. 2015). Over the past few years, the number of dengue cases has been steadily increasing, with significant seasonal and regional variation. Analysis of data from 2000 to 2017 revealed that almost half of the dengue cases occurred during the monsoon (May–August) and the post-monsoon (September–December) seasons (Mutsuddy et al. 2019). Historically, the monsoon has been the primary dengue transmission season in Bangladesh, although the number of dengue cases has increased during the post-monsoon season in recent years (Haider et al. 2021, Hossain et al. 2023).

Bangladesh’s hot and humid weather favors the production of a large variety of mosquito species with more than 123 species listed in 2016 (Bashar et al. 2016, Irish et al. 2016). The most common vectors of dengue virus, *Ae aegypti* and *Ae albopictus,* were first recorded in 1952 (Asir-Ud-Din M 1952) and recent studies in Dhaka showed a higher Breteau index which measures the number of positive containers per 100 households: 30.8 in 1997, 24.6 in 2000, 55.8 in 2011, 28.7 in 2012, and 22.5 in 2013 (Ferdousi et al. 2015, Paul et al. 2018). In 2022, the maximum Breteau index of >50 was recorded for 6 wards of the Dhaka South City Corporation area (Tawsia Tajmim 2022). The pupal index (PI), which measures the number of pupae per 100 houses, was estimated during the monsoon season in several years in Bangladesh: 62.2 in 2011, 153.5 in 2012, and 75.9 in 2013. However, during the dry period, the PI was estimated as 16.7 in 2012 (Paul et al. 2018).

Climate change, including changes in precipitation, temperature, and humidity, as well as rapid unplanned urbanization, were identified as strong indicators of an ecological imbalance that has led to an increase in dengue cases in Bangladesh (Mutsuddy et al. 2019). These changes could eventually extend dengue transmission season year-round, with a chance of outbreaks occurring at any time of the year. Identifying trends and seasonality in dengue cases may aid health authorities and relevant public and private administrations in effectively allocating resources to control the spread of the DENV through vector control. The objectives of our current study were to (i) compare the annual and monthly number of dengue cases and deaths between 2000 and 2022, (ii) identify the overall trend, and seasonality of dengue cases, (iii) quantify the impact of weather parameters on the number of monthly dengue cases, and (iv) forecast the annual number of dengue cases for the next decade.

## Methods

### Data Sources

The current dengue surveillance in Bangladesh is coordinated by the Management Information System (MIS) of the Ministry of Health and Family Welfare of Bangladesh (Haider et al. 2023). The surveillance includes hospitalized patients diagnosed as infected with dengue virus primarily from government hospitals except in the capital city Dhaka, where more than 57 private hospitals are included in addition to 20 public hospitals. Outside the capital city Dhaka, the central district hospital of 64 districts and medical college hospitals are also included in the surveillance system. We collected data on the number of reported dengue cases and deaths from the publicly shared database of the MIS from January 2000 to December 2022. The Ministry of Health and Family Welfare, Bangladesh defines dengue cases based on clinical symptoms (including fever and rash) and/or laboratory tests for IgM or IgG antibodies to DENV and nonstructural 1 protein (NS-1) of DENV (Ahsan et al. 2021).

We used 3-hourly temperature and daily rainfall data from the Bangladesh Meteorological Department (BMD) over the period 2000–2022 from the meteorological station located in Mirpur, Dhaka (Lat 23.46, Lon 90.23). Given Bangladesh’s relatively small land size and moderate climate variation across the country, we focused data solely on the Dhaka station. Furthermore, a substantial proportion of historical dengue cases (>90%) have originated from Dhaka city (Sharmin et al. 2018).

### Procedures

The monthly number of reported dengue cases was used as the primary outcome variable. Two weather variables, temperature and rainfall, were used as the covariates for the regression analysis. In addition, monthly rainfall lagged by 1 or 2 preceding months was also used as predictors for the number of monthly dengue cases.

### Statistical Analysis

We analyzed the monthly dengue cases and meteorological data for the period of 2000–2022. We used 2010 (the median year) to divide the period 2000–2022. Then, we compared the number of dengue cases, deaths, and weather parameters during the 2 decades (2000–2010 and 2011–2022) using a paired sample *t*-test, aimed at comparing trends, developments, and changes between these periods. In the first stage, descriptive analyses were conducted to determine the characteristics of dengue cases and deaths, with mean and interquartile range (IQR) in each month and year calculated for the entire period. Next, we calculated the monthly growth factor (GF) of dengue cases by dividing the number of dengue cases reported in each month by the number of dengue cases reported in the previous month and repeating this process for each month from 2000 to 2022 (Haider et al. 2021). The formula for the GF can be given by


$$\begin{array}{l} GF_{t}=\;\frac{N_{t+1}+1}{N_{t}+1} \end{array}$$

where *N*_*t*_ indicates the number of dengue cases in *t*th month. To avoid the occurrence of zeros in some months, we added 1 to the total number of cases for each month. The distribution of the GF was skewed; therefore, we used the natural log transformation before the data was further examined. However, we have also performed a reverse transformation of the log (GF) values by back-transforming values to the original scale for ease of interpretation (Haider et al. 2021). A monthly GF greater than 1 indicates that the number of dengue cases would be more than the number of dengue cases of the previous month, while a GF less than 1 means that the number of dengue cases in a new month would be less than the previous month. For example, if there are 100 cases in January, the number of dengue cases in February would be 200 when the value of GF is 2.0 or 50 cases when the value of GF is 0.5 in January (Haider et al. 2021).

We performed forecasting using the autoregressive integrated moving average (ARIMA) model. The ARIMA model is a data-driven, exploratory strategy that enabled us to fit a suitable model and forecast values. The ARIMA model consists of autoregressive (*p*) terms, differencing (*d*) terms, and moving average (*q*) operations, and it is denoted as ARIMA (*p*, *d*, *q*) (Kumar and Susan 2020). To select the appropriate autoregressive and moving average orders, the autocorrelation function (ACF) and partial autocorrelation function (PACF) were examined. Additionally, the differencing parameter, represented by “*d*,” indicated the number of nonseasonal differences needed to achieve stationarity (Hasan et al. 2021). We also conducted a Mann–Kendall (M–K) trend analysis to determine possible upward or downward trends (Yue and Pilon 2004). The null hypothesis posits no monotonic trend, while the alternative hypothesis suggests the presence of a trend, which could be positive, negative, or nonnull. We also performed Sen’s slope test to assess variations in annual dengue cases and deaths. The slope greater than 0 indicates an upward trend and less than 0 indicates a downward trend of a given period (Sen 1968).

We then used a time series count generalized linear model (GLM), more specifically, a time series Poisson regression model, to determine whether the meteorological factors were associated with the change in dengue cases over time (Sumi et al. 2021). Monthly dengue cases were utilized as the outcome variable in this model predicted by temperature and rainfall data from the Bangladesh Meteorological Department (BMD). We have estimated the degree-hour of heat generated by the additional temperature each year in Bangladesh. To compare this with the extrinsic incubation period (EIP) of the dengue virus in *Aedes* mosquito, we estimated the degree-hour required to complete the EIP at 26 °C using the mathematical formula [−0.1393 + 0.008 × Temp] presented by Focks et al. (1995). We used the statistical program RStudio, version 3.5.2.2 for the analyses (R Core Team 2022).

## Results

Between 2000 and 2022, DGHS reported a total of 244,246 dengue cases, with an annual mean of 10,619 cases (interquartile range [IQR]: 859.5–5,805.5), including 849 fatal outcomes with a case-fatality ratio (CFR) of 0.35%. Between 2000 and 2010, the mean annual number of dengue cases was 2,216 (IQR: 480–3,182), which increased by 8 times in the following decade (2011–2022) at 18,321 (IQR: 1,405–28,429, *P-value* = 0.22) (Table 1). Between these 2 periods, the mean number of annual deaths due to DENV cases increased by 2.2 times, from 21.2 to 46.6 cases (*P*-value= 0.85). However, the CFR of DENV cases decreased slightly from 0.69% to 0.23% (*P*-value = 0.08) (Table 1).

**Table 1.** Comparison of dengue cases, deaths, and weather parameters between the first (2000–20210) and the recent decades (2011–2022) in Bangladesh

	First decade (2000–2010)	Recent decade (2011–2022)	*P*-value
Mean annual dengue cases (interquartile range [IQR])	2,216.64 (480–3182)	18,321.92 (1405–28429)	0.219
Mean annual dengue deaths (IQR)	21.18 (0.0–28.5)	46.58 (3.0–105.0)	0.853
Mean Case-fatality ratio (±SD)	0.69 (±0.79)	0.23 (±13)	0.08
Mean annual temperature °C (±SD)	26.35 (±0.49)	26.84 (±0.37)	<0.001
Mean annual rainfall in mm (±SD)	2,078.66 (±459.68)	1,764.50 (±448.32)	0.188

**Fig 1.**
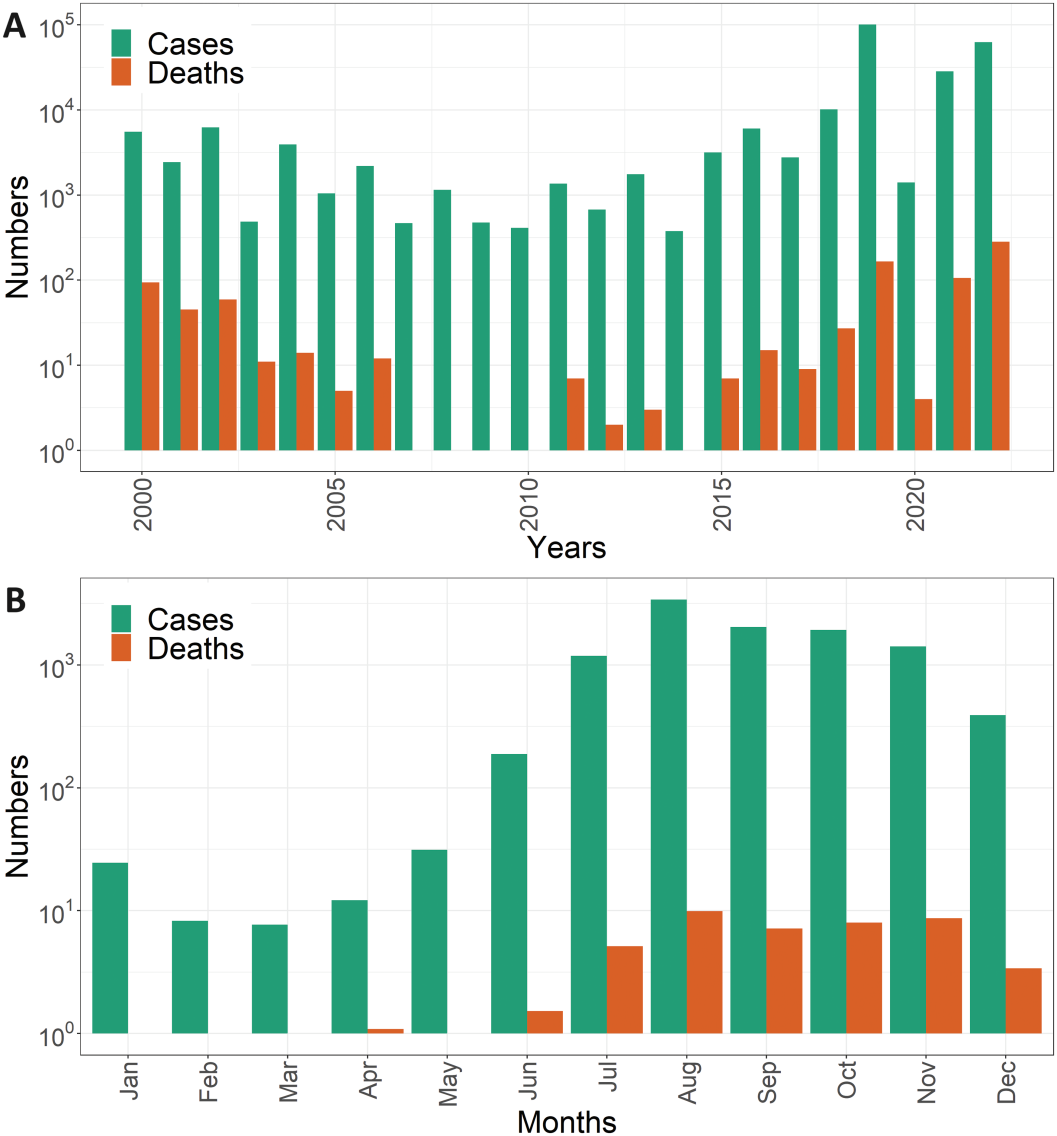
(A) Number of dengue cases and deaths over the period 2000–2022, Bangladesh. (B) Number of monthly dengue cases and deaths recorded in Bangladesh, 2000–2022.

The highest monthly average number of cases was recorded in August (*n* = 3,407 cases) and the lowest was in March (*n* = 6.7 cases) (Fig. 1B). The highest number of annual cases was reported in 2019 with 101,354. The highest number of deaths was recorded in 2022 with 281 deaths, which was 35% of total deaths recorded in the past 23 yr in Bangladesh (Fig. 1). Most (65%, *n* = 550) dengue-related deaths were recorded after 2018 (Fig. 1).

**Fig 2.**
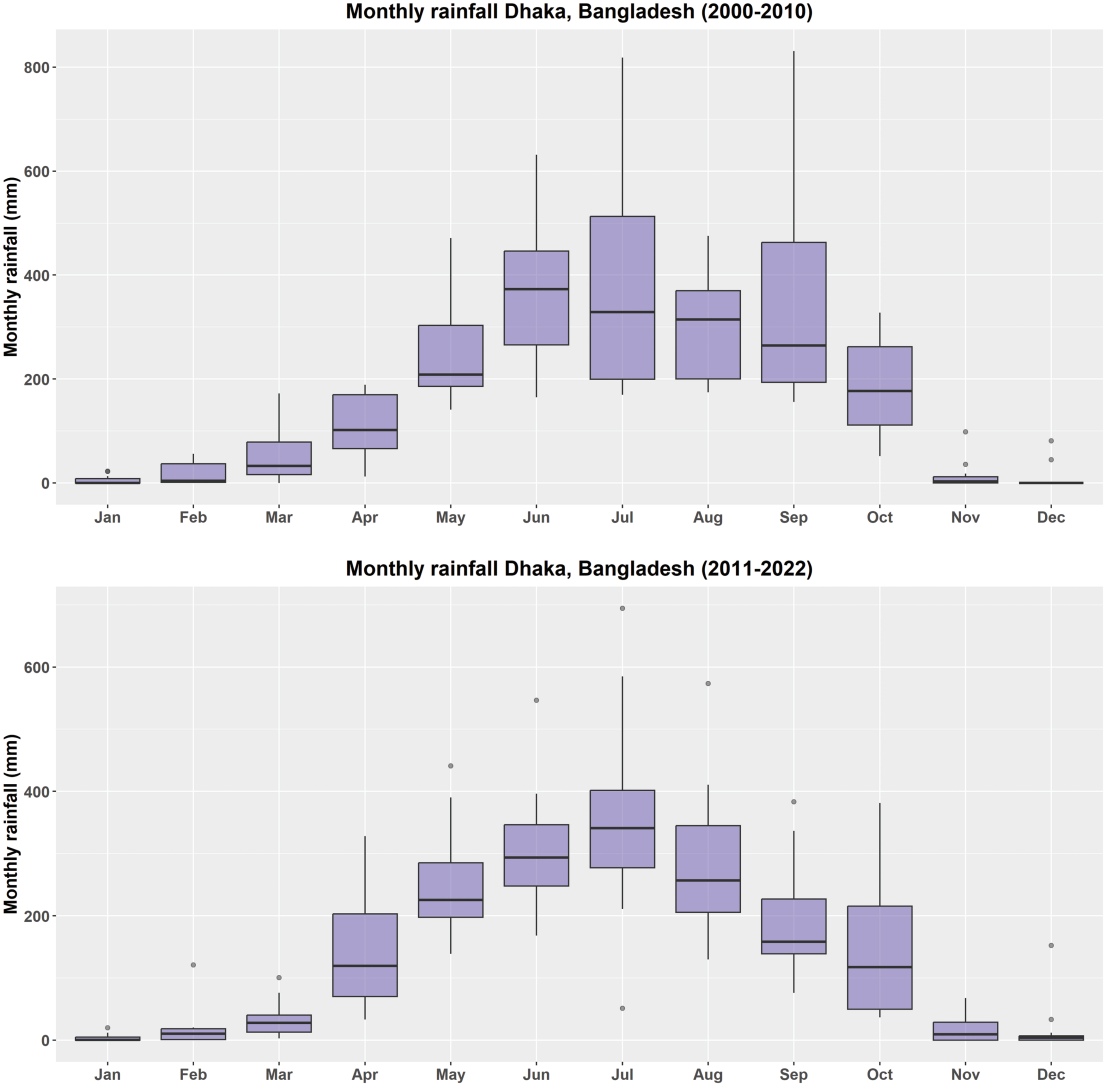
The boxplot compares monthly rainfall in Dhaka city, Bangladesh between 2 decades (2000–2010 vs. 2011–2022). The bottom and top of the box indicate the first and third quantiles, the band inside the box is the median. The dots outside the box are individual outliers. Most of the months in the second decade had outlier rainfall whereas in the first decade, only the cooler months (Nov–Jan) had some extreme rainfall.

**Fig 3.**
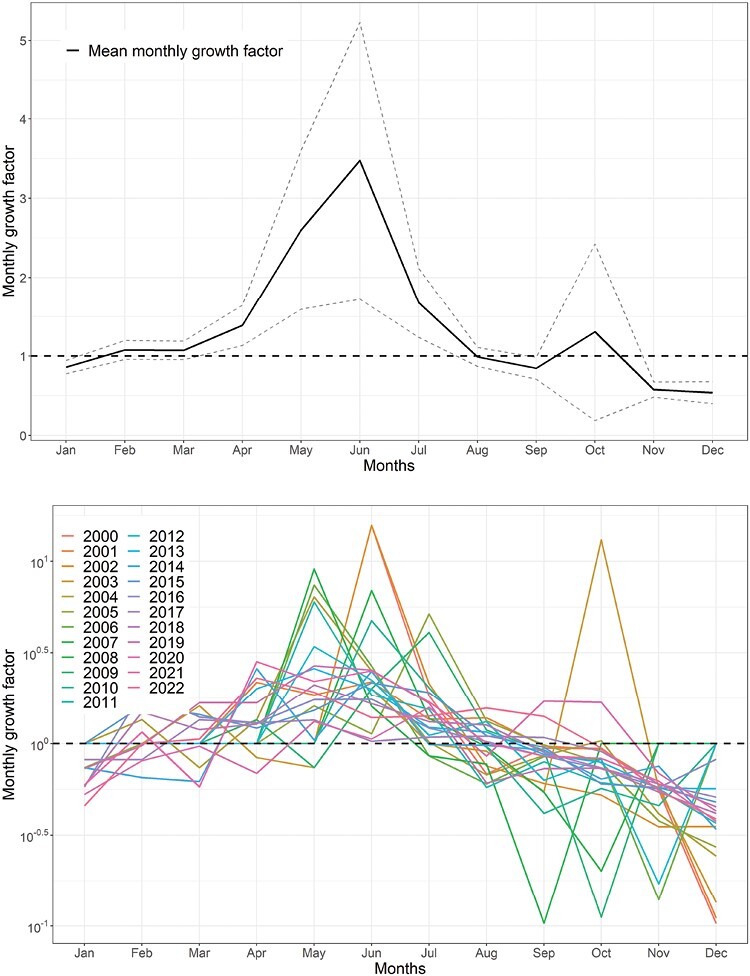
(Top) Mean monthly growth factor for the period of 2000–2022. (Bottom) The monthly growth factor for the individual year 2000–2022. The horizontal dashed line indicates monthly growth factor 1 (the same number of dengue cases in 2 subsequent months).

The average annual temperature was 26.35 °C (SD = 0.49) during the first decade (2000–2010) and 26.84 °C (SD = 0.37) during the recent decade (2011–2022) (Table 1). The increase of 0.49 °C temperature was equivalent to 4,292 degree-h/yr of heat (365 days × 24 h × 0.49 °C). For dengue virus transmission, approximately 349 degree-h of equivalent heat is needed to complete the EIP of DENV in the *Aedes* mosquito at 26 °C (Focks et al. 1995). The annual total rainfall decreased by 314 mm between the 2 decades, from 2078.6 to 1764.5 mm (Table 1), of which 308 mm decreased during the monsoon (July–October) season and only 6 mm decreased during the nonmonsoon period. However, during pre- and post-monsoon season, rainfall (more than third quantile value of monthly rainfall for the decade) increased in the second decade (Fig. 2). The overall mean GF for the number of dengue cases per month was 1.37 (SD = 0.86). However, in 4 months (April–July), the monthly GF was above 1 (lower 95% confidence interval > 1), while for the rest of the months, the monthly GF was less than 1 (95% confidence interval crossed 1). More than 77% (71/92) of months between April and July for the period 2000–2022 had mean monthly GF > 1 compared to only 16% (30/184) of months between August and March of the same period. June had the highest GF with a mean value of 3.47 indicating that cases would be more than 3 times higher in the next month (July). The lowest GF was recorded in December with a mean of 0.54 (95% CI: 0.40–0.69) indicating that cases in January would be nearly halved compared to the number of cases recorded in December (Fig. 3). In the M–K trend analysis, we found a positive trend of reported dengue cases (*P*-value < 0.001 and tau = 0.26). In Sen’s slope test, the slope was 171.67 (95% CI: −46 to 687) with a tau value of 0.26 and *P*-value of 0.14 indicating a nonsignificant upward trend.

 Using the GLM, we estimated the effect of each variable presented as the incidence risk ratio (IRR). The model indicates that dengue cases would rise by 26% with a 1 °C temperature increase. For each additional centimeter of rainfall in the first-lagged month, the number of dengue cases increased by 8% (IRR = 1.08 [95% CI: 1.08–1.09]), and in the second-lagged month increases in cases would be by 17% (IRR = 1. 17 [95% CI: 1. 17–1.18]).

**Fig 4.**
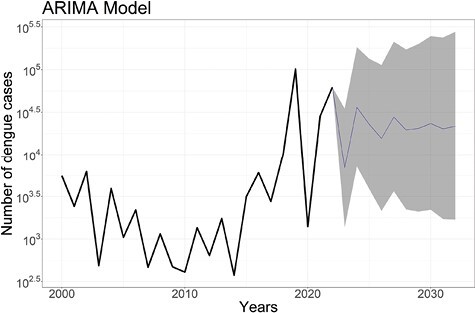
The observed and forecasted number of dengue cases in Bangladesh using the autoregressive moving average (ARIMA) model including a 95% confidence interval.

In the ARIMA model, we detected an increasing trend for the first few years, which then started to decline. However, a strong rise in cases was observed after 2018 except for 2020 (the first year of the COVID-19 pandemic). The forecasted value showed a flat line with reduced variation over time in the number of dengue cases in Bangladesh (Fig. 4).

## Discussion

Dengue is currently an important public health challenge for Bangladesh. Our analysis showed that the number of DENV cases has increased 8 times, deaths have doubled, and the CFR dropped to one-third between the first and second decades of this century. Between these periods, the annual temperature increased by 0.49 °C, and annual rainfall decreased by 314 mm, despite changes in the seasonality of rainfall with unusually early or late rainfall outside the typical monsoon season (July–October) (Haider et al. 2014). The monthly GF remained above 1 for 4 months (April–July) which overlapped the hot and humid period of the year. Monthly mean temperature, monthly first-lagged rainfall, and second-lagged rainfall played a critical role in monthly dengue cases in Bangladesh.

The average increase of 0.49 °C temperature added approximately 4,292 degree-h of equivalent heat per year. This additional heat would favor VBD transmission. For DENV, approximately 349-degree-h equivalent heat is needed to complete the EIP in *Aedes* mosquitoes at 26 °C (Focks et al. 1995). Therefore, the addition of 0.49 °C temperature shortens the duration of the EIP and thus increases the rate of dengue virus transmission. An 8-fold increase in dengue cases is a possible indication of the impact of increases in temperature in the country. Our model identified a significant role of monthly mean temperature, with an additional 1 °C temperature increasing the monthly cases by 26%. Earlier studies showed that for every 1 °C increase in temperature, dengue cases increased by 61% in Australia, 12%–22% in Cambodia, 5% in Vietnam, and 2.6% in Mexico (Soneja et al. 2021). Increasing temperatures can accelerate mosquito population growth and shorten the duration of the EIP of the virus, thereby allowing an increased biting rate and more frequent transmission (Najmul Haider 2018, Couper et al. 2021). Decreased rainfall can increase the risk of dengue, especially in urbanized areas that may have an inadequate and intermittent water supply during drought (Lowe et al. 2021).

Rainfall provides oviposition and larval developmental sites and thereby plays an important role in mosquito population size and pathogen transmission. Although we found a 15% reduction in annual rainfall in the recent decade from the immediate past decade, we detected an increase in rainfall during pre- and post-monsoon seasons, thereby extending the season for mosquitoes and other arthropod vectors. Our model showed that the first- and second-lagged month’s rainfall increased monthly cases by 8% and 17%, respectively. These findings were consistent with earlier studies in Bangladesh that showed that peak dengue cases occurred 2 mo after peak rainfall (Salje et al. 2016) or an additional rainy day per month increased dengue cases by 6% in the succeeding month (Rahman et al. 2020). Similar findings were reported in Vietnam with dengue cases being associated with both first and second-lagged months (Cuong et al. 2011). In the greater part of the capital city Dhaka, there is a shortage of municipal water, and thus people attempt to store municipal water when available as well as rainwater. This might facilitate the production of *Aedes* mosquitoes (Akanda et al. 2020). In Timor-Leste, a 47% increase in dengue cases was recorded with an additional 1-mm seasonal rainfall increase (Wangdi et al. 2018). These findings are biologically plausible as altered precipitation during pre- and post-monsoon allows extended vector seasons facilitating additional human cases (Yuan et al. 2020).

Bangladesh’s dengue season is characterized by hot and wet periods from June to August. This is the period with the highest amount of rainfall facilitating *Aedes* abundance (Haider et al. 2023). The monthly mean GF above 1 for April–June indicates that for each of these months, the number of dengue cases will surpass the previous month. Thus, we suggest starting vector control intervention in April in Bangladesh.

Two large dengue outbreaks occurred in Bangladesh in 2019 and 2022, with both characterized by unusual weather patterns and the occurrence of 2 different DENV serotypes. The 2019 outbreak was characterized by early rainfall of 120 mm in February compared to a historical monthly mean of 20-mm precipitation, along with the introduction DENV-3 (Ahsan et al. 2021). The 2022 outbreak was characterized by the late onset of rainfall, with 297 mm of rainfall in October compared to a monthly mean of 156 mm that may have prolonged the vector transmission season and by the introduction of DENV-4 (Haider et al. 2023 May 18). The introduction this new serotype exposed a largely naive population in a densely populated country like Bangladesh. A large proportion of the population had already been infected with 1 or more serotypes of DENV with more than 80% of people living in Dhaka having antibodies against DENV (Salje et al. 2016). Another study predicted an estimated 40 million people had been infected with DENV nationally, with 2.4 million annual infections (Salje et al. 2019). Thus, any subsequent infections raise the risk of developing severe dengue hemorrhagic fever through antibody-dependent enhancement (ADE) (Teo et al. 2023). The deaths of many people in 2022 when DENV-4 was introduced were probably associated with secondary and/or tertiary DENV infection (Haider et al. 2023).

Our analysis shows that there was a significant monotonic increasing trend of dengue cases in Bangladesh for the period 2000–2022 (M–K trend test); however, the magnitude of the increasing trend was not significant (Sen’s Slope test). This might be due to the large variation of the cases reported in different years. For example, more than 82% of dengue cases (*n* = 202,425) that were recorded in the last 23 yr (2000–2023) were reported in the recent 5 yr (2018–2022). The increase in case reporting in recent years might be a true increase in dengue cases or could be the result of the development of the health care system, improved diagnostic system, and inclusion of more hospitals in the surveillance system in Bangladesh (Haider et al. 2023).

Controlling VBDs in tropical countries where temperatures, humidity, and rainfall remain favorable for mosquitoes during most of the year is a difficult task (Haider et al. 2023). Concerns have been raised over the development of insecticide resistance (Al-Amin et al. 2020, Ahsan et al. 2021) and the failure of developing a successful dengue vaccine (Wang et al. 2017). The prospect of *Wolbachia-*related intervention is still far from being applied on a national scale considering the expenses and associated technicalities. In this situation, an integrated and holistic vector management plan engaging the local communities is key for controlling *Aedes*-borne diseases, especially in resource-limited countries. Regular destruction of mosquito developmental sites and increasing surveillance for detecting active cases are key to limiting dengue virus infections. The development of a municipal water system that would preclude the need to store water is essential to prevent *Aedes* mosquito production. Continuous active surveillance for DENV cases will enable early detection of cases and the location of outbreaks. Public health authorities will be able to identify areas where the disease is spreading, take immediate action to control mosquito populations, isolate infected patients, and implement public awareness campaigns to educate people about preventive measures. Early detection and response can help prevent the further spread of the disease and reduce its impact.

Several weaknesses may have impacted our study. We relied on the reported number of cases from the Ministry of Health and Family Welfare’s website, which mainly relies on passive reporting systems from the selected health facilities in the country (Ahsan et al. 2021). These numbers seem to underestimate the actual number of infections and fever cases. The hospitals included in the surveillance system are only a small fraction of total healthcare facilities in Bangladesh (~5%) where dengue patients can seek healthcare (Haider et al. 2023). A study in Bangladesh based on the national seroprevalence of DENV antibodies predicted an annual infection of 2.4 million people (Salje et al. 2019). Dengue cases similarly are underestimated globally as it is difficult to detect asymptomatic or mild cases that never reach healthcare settings. Although mild cases are missed frequently, severe and fatal cases would likely visit the hospital and thus be counted as numerators in our estimates. Hence, our estimation did not overlook the worst-case scenario, but may have estimated a higher CFR because of the underestimation of the denominators. Another limitation pertains to our exclusive utilization of weather data from the Dhaka station. Given Bangladesh’s relatively small size and the moderate climate variation across the country, we focused our data collection solely on the Dhaka station. However, a substantial proportion of historical dengue cases originated from Dhaka city. We could not use herd immunity data in our model as these data are not available for different serotypes of DENV in Bangladesh. However, earlier studies show that people living in the capital city and larger cities like Chittagong have higher seroprevalence compared to rural areas where the seroprevalence was as low as 3% (Salje et al. 2019). This also illustrates a high risk of ADE through secondary and tertiary infection in large cities. We accept that the increase in dengue cases in the recent decade could be a result of multiple factors that we could not include in the analysis. These factors include the improvement of the healthcare system, which now detects a greater proportion of clinical cases than in the past, the arrival of new serotypes of DENV, and the increased size of the urban population.

## Conclusions

Between the first (2000–2010) and the second decade (2011–2022), dengue cases have increased by 8.3 times, and annual deaths have increased by 2.2 times in Bangladesh. This growth of cases may partly be explained by global warming, with an increase of 0.49 °C annual temperature as well as changes in duration and length of the rainy season. Unusual early or late rain in and beyond the monsoon season likely contributed to extending the length of the dengue transmission season in Bangladesh. The monthly mean temperature and monthly total rainfall of the first-lagged month and second-lagged months showed a large influence on the monthly DENV cases in Bangladesh. The mean monthly GF remained significantly above 1 during April–July, which coincided with the hot and rainy season of the country indicating an earlier vector control would benefit the country. We recommend an integrated and holistic vector management plan engaging local communities in the elimination of mosquito larval habitats and increasing surveillance for detecting active dengue cases. Proactive surveillance, vector control, and community engagement remain essential public health interventions. In the context of climate change, urbanization, trade, and the movement of infected people, there is a need to operationalize the One Health approach to address dengue fever and other VBDs in Bangladesh and beyond.

## Data Availability

All the dengue data presented in this manuscript are publicly available on Bangladesh’s Ministry of Health and Family Welfare’s Directorate General of Health Services website (https://dghs.gov.bd/). The meteorological data were purchased from BMD and are restricted to use for research purposes only and anyone interested in these data can request BMD (https://live3.bmd.gov.bd/).

## Notes

Ethics Statement

This study does not include individual-level data and thus does not require ethical approval. We used publicly available data on Dengue cases and deaths. **Conflict of interest:** The authors declare that they have no conflict of interest.
